# Clinical imaging of primary pulmonary nucleoprotein of the testis carcinoma

**DOI:** 10.3389/fmed.2022.1083206

**Published:** 2023-01-04

**Authors:** Wenpeng Huang, Yongbai Zhang, Qi Yang, Ge Gao, Yongkang Qiu, Liming Li, Lei Kang

**Affiliations:** ^1^Department of Nuclear Medicine, Peking University First Hospital, Beijing, China; ^2^Department of Medical Imaging, Peking University First Hospital, Beijing, China; ^3^Department of Radiology, The First Affiliated Hospital of Zhengzhou University, Zhengzhou, Henan, China

**Keywords:** lung neoplasms, nuclear protein in testis carcinoma, computed tomography, positron emission tomography, clinical imaging

## Abstract

**Objective:**

Primary pulmonary nucleoprotein of the testis (NUT) carcinoma is very rare in the clinic. In this study, the clinicopathological manifestations and imaging features of the primary pulmonary NUT carcinoma were investigated to improve the diagnosis of this disease.

**Methods:**

Six patients with pathologically diagnosed pulmonary NUT carcinoma were analyzed, including three males and three females, aged 19–64 (49.00 ± 16.40) years, with clinical manifestations of cough in two cases, hoarseness in one case, blood in sputum in one case, chest pain in one case, and physical examination findings in one case, with a disease duration of 5 days to 4 months. The clinical and imaging data including CT and PET/CT were retrospectively analyzed. Further literature reviews were analyzed in both pulmonary and extrapulmonary NUT carcinoma cases who performed ^18^F-FDG PET/CT.

**Results:**

Most of the patients with pulmonary NUT carcinomas presented as heterogeneous lobulated masses (83.33%), four cases (66.67%) were located in the upper lobe of the left lung, one case (16.67%) in the middle lobe of the right lung, and one case (16.67%) in the lower lobe of the right lung, with the maximum diameter ranging from 1.30 to 8.90 cm and the median of 3.55 cm, most of them were irregularly shaped, with more lobulated margins and more heterogeneous density (83.33%), and the enhancement was mild. PET/CT showed increased ^18^F-FDG uptake in the lesion and metastatic areas. Both the pulmonary NUT patients in this study and literature reviews showed the SUV_max_ of the tumor ranged from 5 to 40 with an average value of 12.8, whereas that of extrapulmonary lesions had a range of SUV_max_ at 4.5–64.1 and a mean of 13.8.

**Conclusion:**

In patients with central lung masses, rapid disease progression, and poor response to initial treatment, the possibility of NUT cancer should be considered and anti-NUT monoclonal antibody immunohistochemical staining, combined with genetic detection, if necessary, should be performed as soon as possible. CT and PET/CT imaging are essential for the staging, management, treatment response assessment, and monitoring of pulmonary NUT cancer.

## Introduction

Primary nuclear protein in testis (NUT) carcinoma is a rare, aggressive, and malignant epithelial cell tumor characterized by an acquired rearrangement of the gene encoding NUT on chromosome 15q14 (1). NUT carcinoma is rare in clinical practice and arises preferentially in children and adolescents. It is most commonly seen in midline anatomical structures such as the mediastinum, head and neck, previously known as NUT carcinoma, but it can also involve non-midline sites such as the lung, kidney, bladder, and iliac bone (2–4). Due to the lack of characteristic histopathological features, it is frequently missed and misdiagnosed. In recent years, the application of anti-NUT immunohistochemical staining has improved the rate of accurate diagnosis. Primary pulmonary NUT carcinoma is very rare, and clinical diagnosis and differential diagnosis are still challenging for physicians (5). In this paper, we analyzed the clinical and imaging features of pulmonary NUT carcinoma and summarizes the novel treatment options to improve the understanding and awareness of it.

## Methods

### Patient selection and general information

The clinical and imaging data of six patients (three male and three female patients) with pathologically diagnosed pulmonary NUT carcinoma between January 2018 and August 2022 were retrospectively collected and analyzed. Their ages ranged from 19 to 64 years (49.00 ± 16.40) at the time of diagnosis. None of the patients had a history of smoking. There were two patients initially presented with cough, one with hoarseness, one with blood in sputum, one with chest pain, and one revealed by physical examination, and with a disease duration of 5 days to 4 months at diagnosis. Laboratory findings showed elevated alpha-fetoprotein (11.90–31.88 ng/mL) in two cases, elevated neuron-specific enolase (26.60–62.80 ng/mL) in two cases, elevated CA724 (8.15–9.97 U/ml) in two cases, elevated non-small cell lung cancer antigen (11.00–210.00 ng/mL) in two cases, elevated CA199 (343.00 U/ml) in one case, and elevated CA125 (39.30 U/ml) in one case. Chemotherapy was performed in three patients (One patient was treated with “pemetrexed + carboplatin + bevacizumab” and two patients were treated with “paclitaxel + carboplatin powder + sindilizumab”), targeted therapy in two patients and radical surgery combined with chemotherapy in one patient.

### Imaging methods

All patients underwent chest CT examinations. CT scans were obtained by using a GE Discovery 750 HD CT scanner, USA, and were routinely performed from the apex of lung to the base of the diaphragm under inspiration with the following scan parameters: tube voltage, 120 kV; tube current using automatic mA technique; section thickness, 5 mm; matrix, 512 × 512; field of view, 18 cm × 18 cm. And one patient underwent whole-body 2-Deoxy-2-[fluorine-18]-fluoro-D-glucose (^18^F-FDG) PET/CT by using a Biograph TruePoint 64 (6) ring PET/CT instrument from Siemens, Germany, while CT was a 64-slice spiral CT. The HM-20 cyclotron and CFN-100 synthesis module from Sumitomo, Japan, were used to synthesize ^18^F-FDG by fully automated chemical method with automatic clinical quality control (radiochemical purity ≥98%). Patients were injected with ^18^F-FDG intravenously at 0.12 mCi/kg, and imaging was performed 50–60 min after injection.

### Pathologic examination

All pathological specimens were fixed in 4% neutral formaldehyde, routinely dehydrated, embedded in paraffin, stained with hematoxylin-eosin (HE), and observed under a light microscope. Immunohistochemical staining (EnVision method) was performed strictly according to the instructions.

### Image analysis

Images were interpreted by one imaging physician and one nuclear medicine physician both with more than 10 years of experience in diagnosis. They assessed the location (central or peripheral), number, density (homogeneous or not), shape (irregular or regular), size (maximum diameter), margins (lobulated, spiculated, or smooth) of the lesions, analyzed the enhancement patterns of the lesions (defining mild enhancement as an increase in CT value of the lesion after enhancement ≤20 HU, moderate enhancement as an increase ≤40 HU, and marked enhancement as an increase >40 HU), and observed the uptake of ^18^F-FDG in the lesions and the whole body to evaluate the metastasis.

## Results

### Imaging findings

**FIGURE 1:**
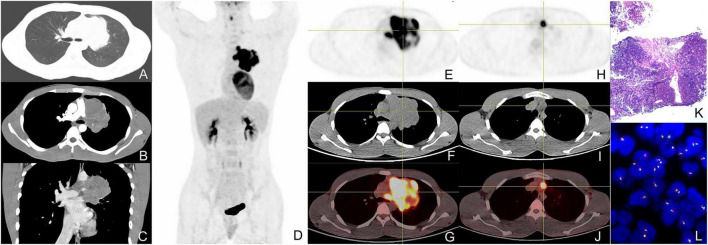
Enhanced CT, ^18^F-FDG PET/CT, and pathological images of a 19-year-old male with left upper lobe central nucleoprotein of the testis (NUT) carcinoma. **(A)** pulmonary window transverse CT demonstrated irregular lobulated paramediastinal mass in the upper lobe of the left lung; **(B)** arterial phase transverse image showed the CT value of the lesion was about 43 HU and tiny vessels could be seen within it. **(C)** Venous phase coronal image showed the CT value of the lesion was about 54 HU, presenting heterogeneous mild enhancement; **(D)** the whole-body maximum intensity projection image of PET/CT showed FDG-avid lesions in the left upper lobe and left hilum of the lung; **(E–G)** tomographic images showed the soft tissue mass with heterogeneous radioactive concentration, and SUV_max_ was about 22.2; **(H–J)** transverse images showed enlarged lymph nodes in mediastinal with radioactive accumulation, and SUV_max_ was about 14.5; **(K)** pathological image (HE staining, magnification × 40); **(L)** fluorescence *in situ* hybridization (FISH) showed the separation of red and green signals in the nuclei of the tumor cells, suggesting the disruption of the NUT gene (magnification × 1,000).

**FIGURE 2:**
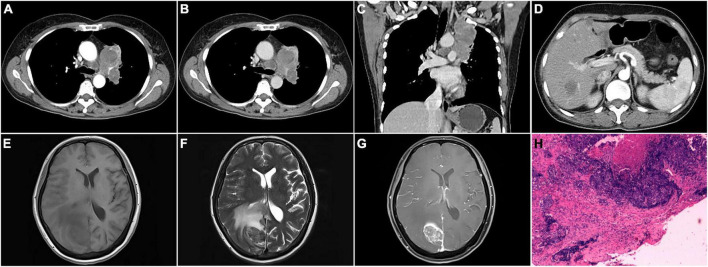
CT, MRI, and pathological images of a 60-year-old female with the left upper lobe central nucleoprotein of the testis (NUT) carcinoma invading pleural, and metastatic to mediastinal lymph node, liver, and brain. **(A)** Arterial phase transverse CT image showed an irregular mass in the left upper lobe with heterogeneous density and displayed enlarged lymph nodes in the mediastinum; **(B)** venous phase transverse image showed heterogeneous mild enhancement of the lesion; **(C)** venous phase coronal image showed the lesion invaded the upper lobe pleura; **(D)** arterial phase transverse image demonstrated metastatic liver lesion; **(E–G)** T1WI, T2WI, and enhanced axial images of brain metastases; **(H)** pathological image (HE staining, magnification × 40).

The CT findings of six patients are summarized in Table 1. Most of them presented as heterogeneous lobulated masses (83.33%), four cases (66.67%) were located in the upper lobe of the left lung, one case (16.67%) in the middle lobe of the right lung, and one case (16.67%) in the lower lobe of the right lung, with the maximum diameter ranging from 1.30 to 8.90 cm and the median of 3.55 cm. Most of them were irregularly shaped, with lobulated margins and heterogeneous density (83.33%), and all tumors showed mild enhancement. Mediastinal lymph node metastasis was observed in five patients (83.33%) and bone metastasis, liver metastasis, and brain metastasis occurred as well. PET/CT was performed in one patient and the lesion with increased glucose metabolism was observed near the hilum in the upper lobe of the left lung, as well as multiple enlarged mediastinal lymph nodes with avid FDG uptake considered as metastasis. In addition, the shape of the left vocal cord was irregular, indicating left recurrent laryngeal nerve was involved (Figures 1, 2).

**TABLE 1:** Chest CT findings of primary lung nucleoprotein of the testis (NUT) carcinoma.

Case	Patient sex	Age/Years	Primary sites	Max diameter/cm	Shape	Margin	Density	Enhancement	Invasion and metastasis
1	M	19	LUL, central	8.8	Irregular	Lobulated	Heterogeneous	Mild enhancement	Recurrent laryngeal nerve invasion; mediastinal lymphadenopathy
2	F	60	LUL, central	8.9	Irregular	Lobulated	Heterogeneous	Mild enhancement	Pleura invasion; mediastinal lymphadenopathy; metastases in liver and brain
3	M	58	RML, central	2.0	Regular	Smooth	Homogeneous	Mild enhancement	Metastases in the right scapula and the 6th left rib
4	F	48	LUL, central	3.3	Irregular	Lobulated	Heterogeneous	Mild enhancement	Mediastinal lymphadenopathy
5	F	45	RLL, peripheral	1.3	Irregular	Lobulated	Heterogeneous	Mild enhancement	Mediastinal lymphadenopathy; multiple metastases in bilateral ribs, thoracic vertebrae and ilium
6	M	64	LUL, peripheral	3.8	Irregular	Lobulated	Heterogeneous	Mild enhancement	Left supraclavicular and mediastinal lymphadenopathy

LUL, left upper lobe; RML, right middle lobe; RLL, right lower lobe.

### Pathologic manifestations

Histopathologic results revealed that the tumors showed diffuse nests of small to medium-sized, completely undifferentiated or poorly differentiated tumor cells with scanty cytoplasm. Partial foci were accompanied by obvious interstitial connective tissue hyperplasia, irregular nuclear contours, prominent nucleoli, and nuclear fission, and some foci showed squamous differentiation and necrosis. The results of immunohistochemistry are shown in Table 2.

**TABLE 2:** Immunohistochemical manifestations of primary lung nucleoprotein of the testis (NUT) carcinoma.

Case	NUT	CK	CK5/6	P63	P40	EMA	CK8/18	SMARCA4	Ki-64
1	+	Partial+	Partial+	/	Partial+	Partial+	+	+	80%
2	+	+	Partial+	/	Partial+	/	Partial+	/	70%
3	+	+	+	+	Partial+	/	−	/	50%
4	+	+	+	/	Partial+	/	+	+	30%
5	+	+	+	Partial+	Partial+	+	Partial+	/	60%
6	+	+	/	Partial+	Partial+	/	+	/	40%

## Discussion

Nucleoprotein of the testis carcinoma is a highly aggressive malignant epithelial cell tumor that accounts for approximately 0.22% of primary lung tumors (7, 8). It harbors unique molecular genetic changes, that is, rearrangements or fusions of the NUT gene on chromosome 15q14 with bromodomain-containing 4 (BRD4), BRD3, nuclear receptor binding SET domain protein 3 (NSD3) or other genes, among which BRD4-NUT fusion is the most common. In 2015, WHO listed it as an independent solid tumor of the chest and classified it as undifferentiated carcinoma (9). NUT carcinoma has no gender preference and can occur at any age, while more prevalent in children and young adults (10), with the median age of lung NUT patients being 30 years (21–68 years) (7, 11). None of the patients in this study had a history of smoking, and the mean age was 49 years, which was higher than that in previous reports. The clinical manifestations of pulmonary NUT carcinoma are closely related to the size and location of the tumor, and the presence of complications or metastasis. And common symptoms include cough, chest pain, hemoptysis, wheezing, dyspnea, fever, weight loss, etc. (7, 12). As for the laboratory tests, it lacks specific tumor markers.

It is still unclear about the specific etiology, pathogenesis, and risk factors of NUT carcinoma (13). The occurrence of it may be related to MYC, P63, and MED24 as well as Wnt, MAPK, and PI3K signaling pathways, but it has no connection with environmental factors and viral infection, such as Epstein-Barr virus and human papillomavirus (7, 14). The tissue origin of NUT carcinoma is likewise unrevealed. Some studies speculate that it may originate from cells derived from primitive neural grooves (15), while others believe that it may arise from pluripotent stem cells (16).

The morphology of lung NUT carcinoma is difficult to distinguish from other poorly differentiated tumors such as Ewing’s sarcoma and small cell lung cancer, and the diagnosis is challenging due to the possibility of misdiagnosis. However, it shows unique alterations in the NUT gene. As for the histological features, small to medium-sized completely undifferentiated or poorly differentiated tumor cells are diffusely arranged in sheets, with sparse, eosinophilic or double eosinophilic cytoplasm, and some foci are accompanied by obvious interstitial connective tissue proliferation, with irregular nuclear contours, most of which are naked nuclei, prominent nucleoli, and frequent nuclear fission (3, 7). What is more, local squamous differentiation can be observed in 33–40% of cases (4, 10), and extensive necrosis sometimes exists. Lung cancer with adenocarcinoma morphology should be excluded from the differential diagnosis of NUT carcinoma because NUT carcinoma is considered a non-glandular epithelioid tumor (13). NUT carcinoma can simultaneously express markers of squamous cell carcinoma and neuroendocrine carcinoma (1, 3), and immunohistochemical detection demonstrates that tumor cells are positive for NUT staining, with a specificity of 100% and a sensitivity of 87% (17). And staining of epithelial markers such as AE1/AE3, CK5/6, P63, P40, and EMA is usually positive (18). The diagnosis relies on molecular tests such as immunohistochemical staining, FISH, and RT-PCR (5), but these methods have not been routinely used in clinical practice, resulting in misdiagnosis or delayed diagnosis of NUT carcinoma.

Chest CT is often used to evaluate the characteristics of lung lesions and abnormalities of lymph nodes, pleura, and adjacent bone. Combined with the results of our study and related literature review, the imaging manifestations of NUT carcinoma are summarized as follows: (1) It was reported that pulmonary NUT carcinoma is mainly located in the right lower lobe (19, 20), while in our group it is mostly located in the left upper lobe, which is inconsistent with previous reports. The tumor is large, mostly central-located, with aggressive and infiltrating growth, irregular shape, and mostly lobulated margins. It can also be manifested as massive pleural effusion, lobar collapse, obstructive atelectasis, and mediastinal widening or displacement (21, 22). (2) The tumor showed low-density soft tissue mass with the low-density necrotic area and occasional calcification in it on plain scan (19), and heterogeneous enhancement on enhanced images. (3) NUT carcinoma can invade the bronchial wall or blood vessels (7, 23), and is frequently associated with ipsilateral mediastinal lymph node metastasis and pleural involvement in the form of pleural thickening or nodules (13, 21), while some cases presenting with supra clavicular, contralateral mediastinal and subcarinal lymph node metastasis. The location of extrathoracic metastasis is mainly bone, with osteolytic destruction manifestation on CT, and liver, adrenal gland, and subcutaneous soft tissue involvement has also been reported (7). However, brain metastasis is rare, and we speculate that it is due to the rapid progression of tumors and the short survival time of patients, which is not enough to develop brain metastasis. Mediastinal and left supraclavicular lymph node metastasis, bone metastasis, and liver metastasis occurred in our patients, and brain metastasis occurred in one patient during disease progression. Therefore, there is no specific metastatic route for lung NUT cancer.

^18^F-FDG PET has been widely used for staging, restaging, and response evaluation of lung cancer. Considering lung NUT cancer is highly aggressive and usually accompanied by symptoms and signs caused by extrathoracic distant metastasis, it is recommended that patients undergo PET/CT examination to comprehensively evaluate whether they have developed other invaded sites, which is useful for the staging, early detection of metastasis, and evaluation of the metabolic and anatomical characteristics of the tumor. We searched the literature in the PubMed and Google Scholar databases using the keywords containing lung, NUT and PET. The PET/CT manifestations of total 18 cases from 2014 to 2022 are summarized in Table 3 (12, 22, 24–29). The most frequently involved lobe is right upper lobe of the lung among these cases (8 in 17) and the mean max diameter of the lung lesions is 5.0 cm (1.4–12.0 cm). The feature of lung NUT on PET/CT is the increased uptake of ^18^F-FDG in the lesion and the metastatic areas. In the reported cases we summarized in Table 3, the SUV_max_ of the tumor could range from 5 to 40 and the mean value is 12.8, and the number of patients with a SUV_max_ of more than 10.0 was up to 10 in these 14 available cases. And the most common metastatic sites are lymph nodes and bones, with FDG-avid presentation on PET/CT as well. We additionally searched for the extrapulmonary NUT for more extensive analysis using the keywords including NUT and PET and screened out the primary lung NUT. Totally 22 cases containing descriptions of PET/CT are included and summarized in Table 4 (30–50). Extrapulmonary sites involved include commonly the mediastinum and nasal sinuses and sometimes the neck and abdomen. The presentation of extrapulmonary lesions on PET/CT is similar to that of pulmonary lesions with a range of 4.5–64.1 and a mean of 13.8. Interestingly, the age of onset of extrapulmonary NUT is significantly younger than that of pulmonary NUT (23.9 ± 4.2 of extrapulmonary lesions vs. 42.7 ± 3.8 of pulmonary lesions, *P* = 0.002).

**TABLE 3:** PET/CT manifestations of primary lung nucleoprotein of the testis (NUT) carcinoma.

Case	References	Patient sex	Age/Years	Primary sites	Max diameter/cm	Margin	SUV_max_	Invasion and metastasis	Management	Prognosis
1	Chang et al. (24)	M	43	LUL, peripheral	1.5	Lobulated	5.1	None	Surgery	Alive at 6 months
2	Chang et al. (24)	M	44	RUL, peripheral	2.1	Lobulated	11.1	Ipsilateral segmental lymphadenopathy.	Surgery + chemotherapy	Alive at 9 months
3	Chang et al. (24)	M	38	RUL, central	3.1	Lobulated	5	Multiple ipsilateral hilar, and paratracheal lymphadenopathy.	Surgery + chemotherapy	Alive at 21 months
4	Chang et al. (24)	F	48	RLL, central	3.1	Lobulated	15.1	Multiple ipsilateral interlobar, hilar, and subcarinal lymphadenopathy.	Surgery + chemotherapy	Alive at 10 months
5	Chang et al. (24)	F	18	LUL, central	4.4	Lobulated	40	Ipsilateral mediastinal lymphadenopathy.	Chemotherapy + RT	Alive at 1 months
6	Chang et al. (24)	M	45	RLL, peripheral	5.2	Lobulated	10.5	Multiple ipsilateral interlobar, hilar, and subcarinal lymphadenopathy.	Chemotherapy + RT	Alive at 35 months
7	Chang et al. (24)	F	41	RUL, peripheral	3.3	Lobulated	5	Multiple ipsilateral interlobar, hilar, and subcarinal lymphadenopathy; ipsilateral diffuse and nodular pleural seeding and effusion.	Chemotherapy	Died at 3 months
8	Chang et al. (24)	M	34	RML, peripheral	2.3	Lobulated	5	Multiple osteolytic bone metastasis.	Chemotherapy + RT	Died at 12 months
9	Chang et al. (24)	M	32	LLL, central	6.5	Lobulated	12.4	Multiple ipsilateral interlobar, hilar, and bilateral mediastinal lymphadenopathy; multiple metastasis in liver, adrenal glands and bones.	Chemotherapy	Died at 3 months
10	Baras et al. (25)	M	39	LLL, central	12	NA	NA, intensely FDG-avid	Confluent mediastinal lymphadenopathy; multiple metastasis in liver and bones	Chemotherapy + RT	Died at 4 months
11	Numakura et al. (26)	M	82	RUL, peripheral	1.4	NA	NA	Ipsilateral hilar and mediastinal lymphadenopathy	Surgery	NA
12	Gupta et al. (27)	M	49	RUL, central	6.6	Lobulated	NA, increased FDG uptake	Paratracheal and generalized mediastinal lymphadenopathy; multiple metastasis in brain and muscles.	Chemotherapy + targeted therapy + RT	Died at 18 months
13	Bair et al. (22)	M	22	RLL, central	NA	NA	>12	Extensive mediastinal and pericardial invasion.	Surgery + chemotherapy	NA
14	Xie et al. (12)	M	23	RUL, central	5.5	NA	10.6	Multiple contralateral hilar, and bilateral mediastinal lymphadenopathy; multiple metastasis in bones.	Surgery + targeted therapy	Died at 1.5 months
15	Xie et al. (12)	M	53	RUL, central	5.4	NA	18.6	Multiple ipsilateral supraclavicular, hilar, and mediastinal lymphadenopathy; multiple metastasis in adrenal glands and bones.	Chemotherapy + targeted therapy	Died at 4.1 months
16	Xie et al. (12)	M	74	LLL, RUL, peripheral	2.8, 5.3	NA	13.6, 18.4	Supraclavicular (R), hilar (L), mediastinal (L) lymphadenopathy.	RT + targeted therapy	Died at 19.5 months
17	Gasljevic et al. (28)	M	47	Left	12	NA	13	Multiple mediastinal and peripheral lymphadenopathy; multiple metastasis in bones, subcutaneous tissue, and pleura.	Chemotherapy	Died at 2 months
18	Cavalieri et al. (29)	F	37	LLL	NA	NA	8.8	Multiple metastases in lung, pleura, and bones	Surgery + chemotherapy + RT + targeted therapy	Died at 2 year

LUL, left upper lobe; RUL, right upper lobe; RLL, right lower lobe; RT, radiation therapy; RML, right middle lobe; LLL, left lower lobe; NA, not available.

**TABLE 4:** ^18^F-FDG PET/CT manifestations of extrapulmonary nucleoprotein of the testis (NUT) carcinoma.

Case	References	Patient sex	Age	Clinical presentation	Primary sites	Max diameter/cm	SUV_max_	Invasion and metastasis	Management	Prognosis
1	Ciftci et al. (30)	M	7	Pallor, coughing, sweating and anorexia	Right hemithorax	NA	5.8	Left cervical, mediastinal, abdominopelvic lymphadenopathy; multiple metastases in liver and bones	Chemotherapy	NA
2	Rutt et al. (31)	F	8	Sore throat, neck swelling and enlarged right tonsil	Right tonsil	4.2	13	Bilateral cervical lymphadenopathy; multiple metastases in bones	Chemotherapy + RT	Died
3	Rosenbaum et al. (32)	M	17	Weight loss, anorexia and chest and right lower extremity pain	Posterior mediastinum	10.6	8.7	Right-side cervical lymphadenopathy; multiple metastases in bones and liver	Chemotherapy	Died at 5 months
4	Polsani et al. (33)	M	2	Fever, sweating, vomiting and abdominal distention	Right upper quadrant of abdomen	NA	NA	Invasion to the left lobe of the liver, pancreatic head and adjacent vessels; metastasis in the right hepatic lobe	Chemotherapy	NA
5	Kawase et al. (34)	M	52	NA	Right hilum	NA	5.3	Right upper and anterior mediastinal lymphadenopathy	Chemotherapy + RT	Alive at 30 months
6	Shaikh et al. (35)	F	29	Excessive tearing of the left eye and headache	Left medial canthus region and nasal cavity	1.5	NA	Multiple bilateral cervical lymphadenopathy	Surgery + chemotherapy	NA
7	Harada et al. (36)	M	28	Coughing and left chest pain	Mediastinum and RML	NA	NA	Multiple ipsilateral hilar, and supraclavicular lymphadenopathy; metastases in bones and right orbital	Chemotherapy + RT	Died at 4.5 months
8	Maur et al. (37)	M	21	Fatigue, fever, and right chest pain	Right anterior upper mediastinal	13.5	NA	Invasion to right lobe of the lung; multiple right supraclavicular, axillary, and mediastinal lymphadenopathy; metastases in subcutaneous areas, peritoneal and retroperitoneal carcinosis and bones	Chemotherapy + RT	Died
9	Perkins et al. (38)	F	2	Fever and coughing	Intrapericardial and midline abdominal mesenteric	7.3	4.5	None	Surgery, chemotherapy + targeted therapy	Died at 11 months
10	Storck et al. (39)	M	9	Fever, vomiting and indigestion	Right sublingual gland	NA	NA	Multiple cervical, supraclavicular, mediastinal lymphadenopathy	Chemotherapy + RT	Alive at 5 year
11	Storck et al. (39)	M	9	Left cheek swelling	Masseter muscle	3	NA	Left-sided submandibular lymphadenopathy	Chemotherapy + RT	Alive at 8 months
12	Newallo et al. (40)	F	64	Coughing, congestion and substernal tightness	Superior mediastinum	NA	NA	None	Chemotherapy	Died at 1 months
13	Sopfe et al. (41)	F	12	Right eye swelling, pain, frontal headaches, right cheek numbness, and nausea.	Right nasal cavity	NA	11	Slight intracranial extension; metastases in bones	Chemotherapy + RT + surgery	Alive at 40 months
14	Joel et al. (42)	F	34	Coughing, hemoptysis and right-side shoulder pain	Right lung, mediastinum, inferior angle of the right scapula	NA	NA	Multiple metastases in bones	Chemotherapy + RT + targeted therapy	NA
15	Vakani et al. (43)	M	44	Headache and dizziness	Sphenoid sinus	NA	NA	Erosion of adjacent bones	NA	NA
16	Mills et al. (44)	M	23	Hoarseness and loss of weight	Hypopharynx	NA	NA	Bilateral cervical lymphadenopathy	Chemotherapy + targeted therapy	Died at 3 months
17	Leeman et al. (45)	F	15	Right-sided nasal congestion, facial pain, and intractable epistaxis	Right sinonasal	5	8	NA	Chemotherapy + RT	Alive at 34 months
18	Cooper et al. (46)	M	7	Abdominal pain, vomiting	Middle mediastinum	NA	10.7	Paratracheal lymphadenopathy	Chemotherapy	NA
19	Crocetta et al. (47)	F	56	Right epiphora, epistaxis, nasal vestibule swelling and nasal obstruction	Right nasal cavity	NA	15.4	Local bone erosion	Surgery + chemotherapy + RT	Died at 6 months
20	Jimenez et al. (48)	F	5	Non-tender left mandibular mass	Left mandible	6	9.4	None	Surgery + chemotherapy + RT	Alive at 21 months
21	Surro et al. (49)	F	23	Coughing and chest pain	Mediastinum and right hilum	NA	9.5	Supraclavicular and upper mediastinum lymphadenopathy; metastases in bones	Chemotherapy	Died at 20 day
22	Huang et al. (50)	F	58	Eye swelling and pain	Bilateral ethmoid and sphenoid sinuses	4.5	64.1	Localized bone destruction in the septal sinus and pterygoid sinus; distant metastases in bones	Surgery + chemotherapy + RT + targeted therapy	Died at 17 months

Through the analysis of our cases and the literature review, we concluded that PET/CT plays a vital role in the diagnosis, staging and treatment monitoring of NUT. The abnormally elevated FDG uptake on PET/CT is beneficial to distinguish between benign and malignant diseases. Rutt et al. (31) reported an 8-year-old girl with a neck mass mimicking tonsillitis. The presentation on CT indicated a tonsillar abscess while the SUV of 13 on PET scans was suggestive of a malignant disease, and the results of histopathological examination confirmed the diagnosis of NUT. Additionally, with the advantages of the whole body scan and available information of tissue function and biological metabolism, PET/CT is a superior imaging technique of choice for detection and staging of NUT, especially for distant metastases (30, 49). PET scans can accurately reveal the most common bony metastases with significant hypermetabolic activity while conventional imaging modalities such as bone scintigraphy will substantially underestimate the degree of bone involvement (7). Rosenbaum et al. (32) reported a patient developing extensive lytic bone metastases at the later stage of treatment. They found that whole-body bone scintigraphy neglected some lesions revealed by PET scans. Moreover, some studies suggested that PET/CT should be used to monitor treatment response and guide the therapy regimen (34, 36). A patient reported by Niederkohr et al. (51) received ^18^F-FDG PET/CT examination for four times after chemotherapy and the PET results clearly demonstrated the process of remission after initial chemotherapy and aggravated disease burden several months later. PET/CT can reveal both the transient chemotherapeutic response prior to disease progression and residual lesion after operation. Furthermore, the decreased uptake in PET at the center of the mass indicates necrosis within the lesion (19, 32, 52), therefore, PET/CT is an excellent imaging modality of choice for directing percutaneous tissue biopsy (22).

It is difficult to distinguish NUT from other pulmonary tumors based solely on PET/CT findings. Previous literature reported that the SUV_max_ of pulmonary squamous cell carcinoma ranged from 3.1 to 30.2 (6), the SUV_max_ of lung adenocarcinoma ranged from 2.2 to 18.1 (53), the SUV_max_ of pulmonary primary lymphoma ranged from 2.58 to 22.6 (54), the SUV_max_ of small cell lung cancer ranged from 3.3 to 29.9 (55). And there is no obvious difference compared with the data for lung NUT we summarized above. Although lack of pathognomonic imaging findings, NUT should be taken into consideration for differential diagnosis with aggressive clinical presentations, exponential interval growth of the tumor and extensive metastasis (22, 33).

To date, there is no standard treatment for primary pulmonary NUT carcinoma and the prognosis is poor, associated with its rapid progression, susceptibility to recurrence, and unsatisfactory treatment outcome, with a median overall survival of 6.7 months (1, 8). If patients can be diagnosed early, surgery remains the treatment of choice for NUT carcinoma and will help improve the prognosis of patients (5). Due to the aggressive growth of the tumor, most patients already have metastases at the time of initial diagnosis and the outcome of the surgery will be unfavorable or the opportunity for surgery has been missed (56). One study reported a case of primary pulmonary NUT in which the progression of the tumor was inhibited by radiation therapy combined with anlotinib (57). It was suggested that approximately 40% of patients initially responded to chemotherapy and radiotherapy, but always relapsed rapidly and did not respond to subsequent therapeutic interventions (5, 13). Chemotherapeutic agents commonly used include vincristine, cyclophosphamide, etoposide, and adriamycin (21). The use of PD-1 inhibitors as second-line therapy has been reported in the literature to further improve survival (7). The overall outcome of primary lung NUT is unsatisfactory, and molecular targeted therapy is being investigated as a promising therapy, and two molecular targeted agents targeting the underlying pathogenic mechanism have emerged: bromodomain and extra-terminal protein inhibitors (BETi) and histone deacetylase inhibitors (HDACi) are applied in clinical trials and have proved efficacy in some patients, both of which can induce NUT carcinoma cell differentiation and inhibit their growth (4). BETis inhibit the function of BRD4-NUT protein, allowing cell differentiation to proceed. One study showed that two patients administered with a novel BET inhibitor (OTX015/MK-8628) had a significantly higher overall survival (19 and 18 months, respectively) than the previously reported median survival of 6.7 months (58). However, variant NUT may be insensitive to the treatment, and therefore identification of the molecular type is necessary for predicting responsiveness to Beti treatment (59). BRD4-NUT oncoprotein binds and activates histone acetyltransferases, resulting in chromatin acetylation and a feed-forward mechanism, and finally leading to tumorigenesis. HDACis can reverse the function of BRD4-NUT and restore normal cellular processes and is expected to be a new therapeutic target for lung NUT (4), but toxicity and side effects are still issues that need to be addressed (14).

## Conclusion

In summary, the possibility of NUT should be considered in patients with central lung masses and rapidly progressive disease, and anti-NUT immunohistochemical staining should be performed as soon as possible, combined with genetic testing if necessary. CT and PET/CT imaging are essential for staging, management, treatment response assessment, and monitoring of pulmonary NUT cancer.

## Data availability statement

The original contributions presented in this study are included in the article/supplementary material, further inquiries can be directed to the corresponding author.

## Ethics statement

This study was reviewed and approved by the Medical Ethics Committee of the First Affiliated Hospital of Zhengzhou University and Peking University First Hospital. The patients/participants provided their written informed consent to participate in this study. Written informed consent was obtained from the individual(s) for the publication of any potentially identifiable images or data included in this article.

## Author contributions

WH: draft the manuscript. YZ: acquisition and analysis of the work and imaging data collection and analysis. QY and GG: imaging data collection and analysis and resources. YQ and LL: formal analysis and resources. LK: supervision and writing—review and editing. All authors met the requirements for authorship for the submitted version and agreed to its submission.

## References

[1] Moreno V Saluja K Pina-Oviedo S. Nut carcinoma: clinicopathologic features, molecular genetics and epigenetics. *Front Oncol.* (2022) 12:860830. 10.3389/fonc.2022.860830 35372003 PMC8966081

[2] Lantuejoul S Pissaloux D Ferretti G McLeer A. Nut carcinoma of the lung. *Semin Diagn Pathol.* (2021) 38:72–82. 10.1053/j.semdp.2021.06.005 34176698

[3] Claudia G Alexandra G. Challenging diagnosis in nut carcinoma. *Int J Surg Pathol.* (2021) 29:722–5. 10.1177/10668969211019532 34106022 PMC8411478

[4] Zhang H Kong W Liang W. Nut midline carcinoma: a rare solid tumour characterized by chromosome rearrangement. *Evid Based Complement Alternat Med.* (2022) 2022:3369895. 10.1155/2022/3369895 35832518 PMC9273375

[5] Liu Y Li Y Ke X Lu Y. The primary pulmonary nut carcinomas and some uncommon somatic mutations identified by next-generation sequencing: a case report. *AME Case Rep.* (2020) 4:24. 10.21037/acr-19-168 33178996 PMC7608724

[6] Zhang M Wang D Sun Q Pu H Wang Y Zhao S Prognostic significance of Pd-L1 expression and (18)F-Fdg Pet/Ct in surgical pulmonary squamous cell carcinoma. *Oncotarget.* (2017) 8:51630–40. 10.18632/oncotarget.18257 28881674 PMC5584275

[7] Li X Shi H Zhang W Bai C He M Ta N Immunotherapy and targeting the tumor microenvironment: current place and new insights in primary pulmonary nut carcinoma. *Front Oncol.* (2021) 11:690115. 10.3389/fonc.2021.690115 34660264 PMC8515126

[8] Bauer D Mitchell C Strait K Lathan C Stelow E Lüer S Clinicopathologic features and long-term outcomes of nut midline carcinoma. *Clin Cancer Res.* (2012) 18:5773–9. 10.1158/1078-0432.Ccr-12-1153 22896655 PMC3473162

[9] Travis W Brambilla E Nicholson A Yatabe Y Austin J Beasley M The 2015 world health organization classification of lung tumors: impact of genetic, clinical and radiologic advances since the 2004 classification. *J Thorac Oncol.* (2015) 10:1243–60. 10.1097/jto.0000000000000630 26291008

[10] French C. Pathogenesis of nut midline carcinoma. *Annu Rev Pathol.* (2012) 7:247–65. 10.1146/annurev-pathol-011811-132438 22017582

[11] Chau N Ma C Danga K Al-Sayegh H Nardi V Barrette R An anatomical site and genetic-based prognostic model for patients with nuclear protein in testis (Nut) midline carcinoma: analysis of 124 patients. *JNCI Cancer Spectr.* (2020) 4:kz094. 10.1093/jncics/pkz094 32328562 PMC7165803

[12] Xie X Wang L Qin Y Lin X Xie Z Liu M Clinical features, treatment, and survival outcome of primary pulmonary nut midline carcinoma. *Orphanet J Rare Dis.* (2020) 15:183. 10.1186/s13023-020-01449-x 32650830 PMC7350189

[13] Xie M Fu X Wang W. Clinicopathological and molecular characterizations of pulmonary nut midline carcinoma. *Cancer Med.* (2021) 10:5757–64. 10.1002/cam4.4096 34409758 PMC8419746

[14] Zhang Y Han K Dong X Hou Q Li T Li L Case report and literature review: primary pulmonary nut-midline carcinoma. *Front Oncol.* (2021) 11:700781. 10.3389/fonc.2021.700781 PMC8435908 34527578

[15] French C. Demystified molecular pathology of nut midline carcinomas. *J Clin Pathol.* (2010) 63:492–6. 10.1136/jcp.2007.052902 18552174

[16] den Bakker M Beverloo B van den Heuvel-Eibrink M Meeuwis C Tan L Johnson L Nut midline carcinoma of the parotid gland with mesenchymal differentiation. *Am J Surg Pathol.* (2009) 33:1253–8. 10.1097/PAS.0b013e3181abe120 19561446

[17] Bishop J. Newly described tumor entities in sinonasal tract pathology. *Head Neck Pathol.* (2016) 10:23–31. 10.1007/s12105-016-0688-7 26830406 PMC4746135

[18] Saiki A Sakamoto K Bee Y Izumo T. Nuclear protein of the testis midline carcinoma of the thorax. *Jpn J Clin Oncol.* (2022) 52:531–8. 10.1093/jjco/hyac033 35325167 PMC9157292

[19] Sholl L Nishino M Pokharel S Mino-Kenudson M French C Janne P Primary pulmonary nut midline carcinoma: clinical, radiographic, and pathologic characterizations. *J Thorac Oncol.* (2015) 10:951–9. 10.1097/jto.0000000000000545 26001144 PMC4443847

[20] Harms A Herpel E Pfarr N Penzel R Heussel C Herth F Nut carcinoma of the thorax: case report and review of the literature. *Lung Cancer.* (2015) 90:484–91. 10.1016/j.lungcan.2015.10.001 26490121

[21] Virarkar M Saleh M Ramani N Morani A Bhosale P. Imaging spectrum of nut carcinomas. *Clin Imaging.* (2020) 67:198–206. 10.1016/j.clinimag.2020.07.025 32866821

[22] Bair R Chick J Chauhan N French C Madan R. Demystifying nut midline carcinoma: radiologic and pathologic correlations of an aggressive malignancy. *AJR Am J Roentgenol.* (2014) 203:W391–9. 10.2214/ajr.13.12401 25247968

[23] Reddy R Woods T Allan R Malhotra P Mehta H Sarkar P Nut (nuclear protein in testis) carcinoma: a report of two cases with different histopathologic features. *Int J Surg Pathol.* (2019) 27:225–9. 10.1177/1066896918796606 30149737

[24] Chang A Kim T Han J Kim T Choi J. Nut midline carcinoma of the lung: computed tomography findings in 10 patients. *J Comput Assist Tomogr.* (2021) 45:330–6. 10.1097/rct.0000000000001133 33661151

[25] Baras A Naidoo J Hann C Illei P Reninger C Lauring J. Rediagnosis of lung cancer as nut midline carcinoma based on clues from tumor genomic profiling. *J Natl Compr Canc Netw.* (2018) 16:467–72. 10.6004/jnccn.2017.7203 29752320

[26] Numakura S Saito K Motoi N Mori T Saito Y Yokote F P63-negative pulmonary nut carcinoma arising in the elderly: a case report. *Diagn Pathol.* (2020) 15:134. 10.1186/s13000-020-01053-4 33176817 PMC7657348

[27] Gupta R Mumaw D Antonios B Anusim N Dhulipalla S Stender M Nut midline lung cancer: a rare case report with literature review. *AME Case Rep.* (2022) 6:2. 10.21037/acr-21-35 35128310 PMC8762379

[28] Gasljevic G Matter M Blatnik O Unk M Dirnhofer S. Nut carcinoma: a clinical, morphological and immunohistochemical mimicker-the role of Rna sequencing in the diagnostic procedure. *Int J Surg Pathol.* (2022) 30:273–7. 10.1177/10668969211047981 34738485 PMC9003774

[29] Cavalieri S Stathis A Fabbri A Sonzogni A Perrone F Tamborini E Uncommon somatic mutations in metastatic nut midline carcinoma. *Tumori.* (2017) 103(Suppl. 1):e5–8. 10.5301/tj.5000685 28967088

[30] Ciftci E Demirsoy U Anik Y Gorur G Corapcioglu F Demir H. Staging and evaluation of neoadjuvant chemotherapy response with ^18^F-Fdg Pet/Ct in nut-midline carcinoma in a child: a case report and review of the literature. *Rev Esp Med Nucl Imagen Mol.* (2015) 34:53–5. 10.1016/j.remn.2014.08.007 25304847

[31] Rutt A Poulik J Siddiqui A Konski A Kalaf M Madgy D Nut midline carcinoma mimicking tonsillitis in an eight-year-old girl. *Ann Otol Rhinol Laryngol.* (2011) 120:546–9. 10.1177/000348941112000810 21922980

[32] Rosenbaum D Teruya-Feldstein J Price A Meyers P Abramson S. Radiologic features of nut midline carcinoma in an adolescent. *Pediatr Radiol.* (2012) 42:249–52. 10.1007/s00247-011-2288-8 22057302

[33] Polsani A Braithwaite K Alazraki A Abramowsky C Shehata B. Nut midline carcinoma: an imaging case series and review of literature. *Pediatr Radiol.* (2012) 42:205–10. 10.1007/s00247-011-2272-3 22033856

[34] Kawase T Naka G Kubota K Sakashita B Takeda Y. Nut midline carcinoma in elderly patients: usefulness of 18f-Fdg Pet/Ct for treatment assessment. *Clin Nucl Med.* (2015) 40:764–5. 10.1097/rlu.0000000000000795 25899594

[35] Shaikh F Pagedar N Awan O McNeely P. Sinonasal nut-midline carcinoma - a multimodality approach to diagnosis, staging and post-surgical restaging. *Cureus.* (2015) 7:e288. 10.7759/cureus.288 26244120 PMC4523209

[36] Harada Y Koyama T Takeuchi K Shoji K Hoshi K Oyama Y. Nut midline carcinoma mimicking a germ cell tumor: a case report. *BMC Cancer.* (2016) 16:895. 10.1186/s12885-016-2944-3 27855672 PMC5114840

[37] Maur M Toss A Dominici M Frassoldati A Corradini P Maiorana A Impressive Response to dose-dense chemotherapy in a patient with nut midline carcinoma. *Am J Case Rep.* (2015) 16:424–9. 10.12659/ajcr.893879 26140332 PMC4492486

[38] Perkins C Pucar D McDonough C Williams H. Nuclear protein in testis midline carcinoma presenting in an infant as a pericardial mass with staging by (18)F-fluorodeoxyglucose-positron emission tomography/computed tomography. *World J Nucl Med.* (2017) 16:247–50. 10.4103/1450-1147.207284 28670187 PMC5460312

[39] Storck S Kennedy A Marcus K Teot L Vaughn J Gnekow A Pediatric nut-midline carcinoma: therapeutic success employing a sarcoma based multimodal approach. *Pediatr Hematol Oncol.* (2017) 34:231–7. 10.1080/08880018.2017.1363839 29040054

[40] Newallo D Shogbesan G Chataigne M Hall L. Metastatic nut midline carcinoma. *Open J Clin Med Images.* (2022) 2:1068.

[41] Sopfe J Greffe B Treece A. Metastatic nut midline carcinoma treated with aggressive neoadjuvant chemotherapy, radiation, and resection: a case report and review of the literature. *J Pediatr Hematol Oncol.* (2021) 43:e73–5. 10.1097/mph.0000000000001860 32555032

[42] Joel S Weschenfelder F Schleussner E Hofmann G Weschenfelder W. Nut midline carcinoma in a young pregnant female: a case report. *World J Surg Oncol.* (2020) 18:290. 10.1186/s12957-020-02065-6 33160369 PMC7648955

[43] Vakani P Maheshwari J Maheshwari M Shah B. Sinonasal nut midline carcinoma: a new histological entity. *Indian J Pathol Microbiol.* (2020) 63:103–5. 10.4103/ijpm.Ijpm_373_19 32031134

[44] Mills A Lanfranchi M Wein R Mukand-Cerro I Pilichowska M Cowan J Nut midline carcinoma: a case report with a novel translocation and review of the literature. *Head Neck Pathol.* (2014) 8:182–6. 10.1007/s12105-013-0479-3 23912933 PMC4022924

[45] Leeman R Pinkney K Bradley J Ruiz R DuBois S French C Nut carcinoma without upfront surgical resection: a case report. *J Pediatr Hematol Oncol.* (2021) 43:e707–10. 10.1097/mph.0000000000001865 32555033 PMC8887700

[46] Cooper K Hull N Horst K Kolbe A Zingula S Thacker P. Nut carcinoma of the thorax in a 7-year-old child. *Radiol Case Rep.* (2022) 17:1549–53. 10.1016/j.radcr.2022.01.077 35282323 PMC8914253

[47] Crocetta F Botti C Fornaciari M Castellucci A Murri D Santandrea G Sinonasal nut carcinoma: delayed diagnosis due to the Covid-19 pandemic and a review of the literature. *Head Neck Pathol.* (2021) 15:1409–14. 10.1007/s12105-021-01311-x 33686584 PMC7970807

[48] Jimenez C Stanton E Kondra K Nickels E Jacob L Shah R Nut carcinoma of the mandible in a child: case report and systematic review. *Int J Oral Maxillofac Surg.* (2022): [Epub ahead of print]. 10.1016/j.ijom.2022.07.002 35868909

[49] Surro A Al Tarhuni M Al-Katib S. Nut carcinoma resulting in svc syndrome. *Clin Pulm Med.* (2020) 27:54–7. 10.1097/cpm.0000000000000339

[50] Huang W Gao G Qiu Y Yang Q Song L Chen Z Multimodality imaging and treatment of paranasal sinuses nuclear protein in testis carcinoma: a case report. *World J Clin Cases.* (2022) 10:12395–403. 10.12998/wjcc.v10.i33.12395 36483827 PMC9724541

[51] Niederkohr R Cameron M French C. Fdg Pet/Ct imaging of nut midline carcinoma. *Clin Nucl Med.* (2011) 36:e124–6. 10.1097/RLU.0b013e31821c9a23 21825839

[52] Bruzzi J Munden R. Pet/Ct imaging of lung cancer. *J Thorac Imaging.* (2006) 21:123–36. 10.1097/00005382-200605000-00004 16770229

[53] Lee Y Chung J Kim S Kim T Lee K. Adenosquamous carcinoma of the lung: Ct, Fdg Pet, and clinicopathologic findings. *Clin Nucl Med.* (2014) 39:107–12. 10.1097/RLU.0b013e3182952c2d 23751831

[54] Peng Y Qi W Luo Z Zeng Q Huang Y Wang Y Role of 18f-Fdg Pet/Ct in patients affected by pulmonary primary lymphoma. *Front Oncol.* (2022) 12:973109. 10.3389/fonc.2022.973109 36185301 PMC9515576

[55] Choi E Park M Im J Chung Y Oh J. Prognostic value of (18)F-Fdg Pet/Ct metabolic parameters in small cell lung cancer. *J Int Med Res.* (2020) 48:300060519892419. 10.1177/0300060519892419 31880209 PMC7607737

[56] Baumgartner K Lauer U Horger M Zender L Kloth C. [Nuclear protein in testis (Nut) midline carcinoma]. *Rofo.* (2020) 192:303–6. 10.1055/a-1026-6561 31830767

[57] Jiang J Ren Y Xu C Lin X. Nut midline carcinoma as a primary lung tumor treated with anlotinib combined with palliative radiotherapy: a case report. *Diagn Pathol.* (2022) 17:4. 10.1186/s13000-021-01188-y 34996489 PMC8742416

[58] Stathis A Zucca E Bekradda M Gomez-Roca C Delord J de La Motte Rouge T Clinical response of carcinomas harboring the Brd4-nut oncoprotein to the targeted bromodomain inhibitor Otx015/Mk-8628. *Cancer Discov.* (2016) 6:492–500. 10.1158/2159-8290.Cd-15-1335 26976114 PMC4854801

[59] Stevens T Morlote D Xiu J Swensen J Brandwein-Weber M Miettinen M Nutm1-rearranged neoplasia: a multi-institution experience yields novel fusion partners and expands the histologic spectrum. *Mod Pathol.* (2019) 32:764–73. 10.1038/s41379-019-0206-z 30723300 PMC8194366

